# Could postoperative pulmonary oedema be attributed to the use of neostigmine?

**DOI:** 10.4103/0019-5049.72659

**Published:** 2010

**Authors:** Mukul C Kapoor

**Affiliations:** Department of Anaesthesiology, Command Hospital (CC), Lucknow, Uttar Pradesh, India

Sir,

I read with interest the case report by Raiger *et al*,[1] titled “Non-cardiogenic pulmonary oedema after neostigmine for reversal: A report of two cases.” The authors had described two cases of pulmonary oedema after routine surgeries. One patient developed it after tracheal extubation and required a very short period of mechanical ventilation. A laryngeal pack was used in this patient. The other patient developed it before tracheal extubation and required a relatively longer period of mechanical ventilation. This patient was reported to have difficulty in breathing and hence tracheal extubation was delayed.

Post-obstructive pulmonary oedema is caused by significant fluid shifts resulting from changes in intrathoracic pressure[2] Negative intrathoracic pressure generated, when a patient attempts to inspire against a closed glottis or obstructed airway, leads to increase in venous return and a consequent rise in pulmonary venous pressure. This leads to a hydrostatic gradient with fluid moving from high pressure (pulmonary venous system) to low pressure (pulmonary interstitium and airspaces)[3] The negative intrathoracic pressure, along with the resultant hypoxia, also depresses the cardiac output by increasing myocardial wall stress and systemic vascular resistance, which increases the pulmonary venous pressure further.[4] Laryngospasm has been reported to be the cause in >50% of cases, whilst other causes include tracheal secretions, hiccups, and biting the endotracheal tube.

Drug-induced non-cardiogenic pulmonary oedema may be due to a pulmonary venoconstriction, capillary leak syndrome, intravascular fluid volume overload, and/or reduced serum oncotic pressure.[5] Drugs known to cause the above have been enumerated by Reed and Glauser.[5] Neostigmine has never been reported to cause pulmonary oedema in contemporary literature.

Millions of anaesthetics have been delivered with the use of neostigmine but development of postoperative pulmonary oedema has not been attributed to it. It would be too farfetched to attribute the occurrence of pulmonary oedema, in the two cases reported, to the use of neostigmine, without clear evidence or a viable argument.
